# Diabetes Mellitus Does Not Predict Discharge in Hospitalized Patients With Acute Pyelonephritis: A Study From Karachi, Pakistan

**DOI:** 10.7759/cureus.11024

**Published:** 2020-10-18

**Authors:** Furrukh Omair Syed, Fazal U Rehman, Imrana Amin, Syed Ahsan Ali, Bakhtawar J Rind, Bilal Ahmed

**Affiliations:** 1 Medicine, Aga Khan University Hospital, Karachi, PAK; 2 Medicine, Patel Hospital, Karachi, PAK; 3 Internal Medicine, Aga Khan University Hospital, Karachi, PAK; 4 Medicine, Jam Ghulam Qadir Hospital Hub, Karachi, PAK; 5 Medicine, Aga Khan University, Karachi, PAK

**Keywords:** diabetes, pyelonephritis, diabetes mellitus, urinary tract infections

## Abstract

Introduction

The incidence of acute pyelonephritis (APN) in the diabetic population is comparatively higher and tends to be more complicated, with serious outcomes. Although complicated pyelonephritis (PN) needs hospital admission and intravenous antibiotics, the magnitude of hospital stay due to comorbidities is limited. This study's aim was to assess the impact of diabetes mellitus on length of hospital stay among patients with PN.

Methods

We did a retrospective data review of 520 randomly selected hospitalized patients of PN from March 2015 to December 2019 from a tertiary care center. Electronic medical records were used for identifying medical conditions through ICD-10 coding. Length of stay (LOS) was categorized as < five days and ≥ five days. Chi‐squared tests were used to compare categorical parameters. Logistic regression models were used for multivariate analyses.

Results

The study included 520 patients with PN; 194 (37.3 %) men and 326 (62.7%) women. Overall, there were 353 (67.8 %) and 167 (32.1 %) patients with LOS < five and ≥ five days respectively. Most of the patients had lower urinary tract symptoms (90%); among them, the majority (92%) were discharged within five days. Likewise, half of the patients had diabetes (51.2); among them, 53% were discharged after five days. Older age (OR:1.7, 95%CI: 1.1 - 2.6), upper urinary tract symptoms (OR:1.6, 95%CI: 1.1 - 2.4), lower urinary tract symptoms (OR:1.9, 95%CI: 1.1 - 3.5), creatinine greater than 1.5 mg/dl (OR:1.6, 95% CI: 1.1 - 2.4) was positively associated with LOS ≥ 5 days after adjusting for other covariates. Diabetes mellitus was not found to be associated with LOS ≥ 5 days (OR: 0.9, 95%CI: 0.8 - 1.5).

Conclusion

In patients with acute PN, diabetes mellitus is not independently associated with prolonged hospital stay beyond five days.

## Introduction

Globally 10.5 million to 25.9 million cases of pyelonephritis (PN) are diagnosed annually, out of which 459,000 to 1,138,000 cases are reported from the United States only [1]. Given the higher prevalence of risk factors associated with PN, this disease may greatly affect countries from the low and middle-income regions; however, the exact estimates are limited.

Conventionally, acute PN (APN) used to require hospital admission and intravenous antibiotics. However, the recent guideline recommends that mild PN may not require hospitalization and can be treated with oral antibiotics at home. Nevertheless, complicated PN still requires hospitalization for treatment, which has both clinical and economic consequences [2].

Although the literature on medical conditions that predicts PN is well-known, data on the impact of chronic diseases that impact the length of stay among patients with PN is not well-reported. Given that the incidence of acute PN in the diabetic population is comparatively higher, i.e., 12.6% as compared to non-diabetic patients [3-5], the severe clinical manifestations of PN, such as papillary necrosis, emphysematous PN, and bacteremia, were more frequently observed in diabetic patients [6]. In addition, it has been observed in clinical practice that diabetes mellitus generally results in complicated PN and recurrent hospitalization and increases mortality [7]. However, it is not known whether it has any impact on the length of stay (LOS). Our study aims to address this gap and find the association between diabetes mellitus and length of stay in patients with acute PN needing hospital care.

## Materials and methods

Study design and setting

We conducted a retrospective study in hospitalized patients of PN from March 2015 to December 2019. The study was reviewed and approved by our institutional review board.

The study was conducted in a tertiary care academic facility in Karachi, Pakistan. With an annual in-patient census of 6000-8000, approximately 9.63% of patients are being diagnosed as PN [8].

Study subjects

Patients who were 16 years of age or older, with a clinical diagnosis of APN needing intravenous antibiotic therapy were enrolled in the study. Records of the cases were retrieved through codes using International Classification of Disease (ICD) 10 Clinical Modification (CM) N-10 for APN and ICD 10 CM E-10 for diabetes mellitus. The lab results were reviewed through the online portal and patient care services. Of these patients, we included only those patients with pyuria (the presence of 10 or more leukocytes per high power field in the centrifuged specimen), fever (body temperature >38 OC), and lumbar tenderness.

Quantitative urine cultures were performed using a standard technique using Columbia blood and CLED (Cystine-Lactose-Electrolyte-Deficient) agar plates (BioMerieux, Marcy 1’Etoile, France) incubated for 18 to 24 hours at 36°C. Isolated were identified using the analytical profile index (API) system and the Vitek2 automated system (BioMerieux). Susceptibilities were identified using the disk diffusion method or the Vitek 2 system, according to the breakpoints recommended by the Clinical and Laboratory Standard Institute [9].

We also included patients with only pyuria and lumbar tenderness if they had received any antipyretic medication within the last four hours. Exclusion criteria encompassed the following medical conditions: pregnancy, renal stones, severe sepsis, renal tumors, polycystic kidneys, or severe hydronephrosis. Patients getting immunosuppressant medications (steroids, calcineurin inhibitors, anti-metabolites, or cytotoxic agents) were also excluded.

Measures and outcomes

The standardized extraction of demographic and clinical data from electronic medical records was performed by trained data abstractors. The clinical information abstracted included age, co-morbidities, including diabetes mellitus and hypertension, physical findings like fever and costovertebral angle tenderness, and length of hospital stay.

Continuous variables were dichotomized: age <50 years and >50 years. Serum creatinine < 1.5 mg/dl and ≥ 1.5 mg/dl. The main outcome measure was LOS as < five days and ≥ five days.

Data analysis

Continuous data were briefed as mean and standard deviations. Categorical data were summarized as percent frequency of occurrence. Correlation between predictor variables (patient demographics and clinical characteristics) and LOS were analyzed by univariate and multivariate methodologies. For determining the characteristics associated with LOS (> 5 days), stepwise multivariate logistic regression models based on a maximum likelihood estimate method were applied. The crude and adjusted odds ratios (aOR) and 95% confidence intervals (95% CI) are presented. Analyses were done using the Statistical Package for the Social Sciences (SPSS) for Windows 10 (SPSS Inc, Chicago, IL).

## Results

Table 1 shows the characteristics of PN patients included in the cohort. During the study period, 520 patients with APN were enrolled. Of these 520 patients, 194 (37.3%) were male, whereas 364 (62.7%) were females. There were 267 (51.3%) APN patients who had diabetes, whereas 253 were non-diabetic. Altogether, 48.8% patients were hypertensive; half of them were discharged within five days. More than half of the patients (51.2%) discharged after five days had creatinine higher than 1.5 mg/dl. There were about 68.9% of patients who had upper urinary tract symptoms while 91.8% had presented with lower urinary tract symptoms only. The results of the main analysis from the logistic regression model examining the characteristics associated with LOS (< 5 versus ≥ 5 days) are presented in Table 2. Diabetes mellitus was not found to be independently associated with LOS ≥ 5 days (OR: 0.9, 95% CI: 0.8 - 1.5). Other covariates for which the model was adjusted, including age (OR: 1.7, 95% CI: 1.1 - 2.6), upper (OR: 1.6, 95% CI: 1.1 - 2.4) and lower urinary symptoms (OR: 1.9, 95% CI: 1.1 - 3.5), and medical comorbidities, including higher creatinine (OR: 1.6, 95% CI: 1.1 - 2.4), were found to be associated LOS (≥ 5 days). However, gender (OR: 1.1, 95%CI: 0.6 - 1.5), fever (OR: 1.2, 95%CI: 0.8 - 1.7), hypertension (OR: 0.9, 95%CI: 0.6 - 1.3), and total leucocyte count (TLC) (OR: 1.0, 95%CI: 0.9 - 1.0) were not found to be associated with the outcome.

**Table 1:** Sociodemographic and clinical characteristics of APN patients admitted in a tertiary care hospital (Total=520) APN: acute pyelonephritis; DM: diabetes mellitus; HTN: hypertension; E. coli: Escherichia coli

	Total		Length of stay					Crude Estimates	
	N	%	<5 days		>5 days			OR	95%CI
Age in years									
<50 years	153	29.4%	116	32.9%	37	22.2%		1	
>50 years	367	70.6%	237	67.1%	130	77.8%		1.7	1.1-2.6
Gender									
Female	326	62.7%	222	62.9%	104	62.3%		1	
Male	194	37.3%	131	37.1	63	37.7%		0.9	0.6-1.4
URINARY TRACT SYMPTOMS									
Upper									
Negative	146	28.1%	58	34.7%	88	24.9%		1	
Positive	374	71.9%	109	65.3%	265	75.1%		1.6	1.1-2.3
Lower									
Negative	52	10%	23	13.8%	29	8.2%		1	
Positive	468	90%	144	86.2%	324	91.8%		1.7	0.9-1.3
Fever									
No	204	39.2%	134	38%	70	42%		1	
YES	316	60.8%	219	62%	97	58.1%		1.1	0.8-1.7
DM									
No	253	48.7%	175	49.6%	78	46.7%		1	
YES	267	51.3%	178	50.4%	89	53.3%		1.1	0.7-1.6
HTN									
No	266	51.2%	180	51%	86	51.5%		1	
YES	254	48.8%	173	49%	81	48.5%		0.9	0.6-1.4
Creatinine (mg/dl)									
<1.5	298	57.3%	217	61.5%	81	48.5%		1	
>1.5	222	42.7%	136	38.5%	86	51.5%		1.6	1.1-2.4
Urine Culture									
E. coli (All types)	231	44.4%	157	44.5%	74	44.3%		N/A	
Gram-negative (Except E. coli)	244	46.9%	168	47.6%	76	45.5%			
Gram-positive	17	3.3%	12	3.4%	5	3%			
Candida species	28	5.4%	16	4.5%	12	7.2%			

**Table 2:** Association of APN with length of stay in hospitalized patients APN: acute pyelonephritis; TLC: total leucocyte count

	Length of Stay	95%CI	
	a OR		
AGE			
<50 years	1		
>50 years	1.7		1.1-2.6
GENDER			
Female	1		
Male	1.1		0.6-1.5
URINARY TRACT SYMPTOMS			
Upper			
Negative	1		
Positive	1.6		1.1-2.4
Lower			
Negative	1		
Positive	1.9		1.1-3.5
FEVER			
No	1		
YES	1.2		0.8-1.7
DIABETES MELLITUS			
No	1		
YES	0.9		0.8-1.5
HYPERTENSION			
No	1		
Yes	0.9		0.6-1.3
Creatinine (mg/dl)			
<1.5	1		
>1.5	1.6		1.1-2.4
TLC	1.0		0.9-1.0

## Discussion

This study investigated the potential differences in the clinical and microbiological features of diabetic PN patients with a length of stay. The results showed that diabetes mellitus (DM) was not associated with a prolonged length of stay in hospitalized patients with APN. This is in contrast to the higher odds ratios ranging from 1.7 to 4.7 returned by the previous studies [10-11]. A possible explanation for these null findings could be that the older studies had more complicated cases and a lower number of sample sizes (111 and 388 vs 520 in our study) [12]. Another plausible difference is in the difference of the operational definition of the length of stay, as earlier studies took the lengthier cut off for the duration of hospitalization. However, with the feasible use of outpatients parenteral antibiotic therapy (OPAT) in PN, LOS has shortened to a mean duration of three days [13], thus this study has set a five-day cutoff for complicated cases of PN who were hospitalized, which depicts a real-world scenario. Usually, uncomplicated cases have even shorter stays.

Advanced age is considered to be an independent complicating factor of PN [14]. It is easily identified during the initial presentation of PN and can be used for categorizing the PN or forecasting the prognosis. Most of our studied population was above 50 years old (70%), with a mean age of 57.3 years. After adjusting for confounders, our study showed higher odds of delayed discharge as compared to younger-age group patients. As the elderly are known to have a higher number of comorbid conditions and poor immune mechanisms, it is plausible to link advanced age with poor prognosis in APN patients. These findings are in accord with most of the published data on APN, mandating it to be considered as a significant risk for complications in PN [14-15].

Our clinical data established that the frequency of both upper and lower urinary tract symptoms and signs vary significantly, as of all the diagnosed APN cases, 41% did not have any upper tract symptoms. Therefore, symptomatic patients may exhibit an atypical presentation of clinical symptoms/ signs, that it had a prolonged hospital stay as compared to those who presented without upper or lower tract symptoms. Occult PN is an easily missed entity and accounts for a small portion of all PN admissions [16]. Asymptomatic mildly symptomatic patients usually get an early discharge on oral antibiotics.

In the absence of overt complications, PN patients usually discharge within five days of hospitalization [17]. Our study showed 51.3% had less than five days of stay in the hospital with a mean length of stay of 5.36 days. Recently, literature increasingly uses the shorter duration of hospital stay to gauge the quality of care [17-18].

The present study found no association between gender, hypertension, high fever (≥ 39°C or even ≥ 40°C), or a high white blood cell (WBC) count (>12,000 /Ul or even >20,000/uL) and discharge, which advocates that high fever and a high WBC count do not influence discharge plan. We infer that these differences are attributable to the different study populations. Earlier studies are conducted commonly using intravenous agents with a prolonged duration of antibiotics course, which, at times, warrants hospital stay for the ease of intravenous delivery and course completion under monitoring.

Nowadays, a newer version of oral fluoroquinolones and cephalosporin are proven to improve bioavailability and effectiveness, which can be safely taken at home [19]. The clinician feels more confident to send the patient home with a stable infection after an initial response to the treatment within a few days.

Our study findings need to be interpreted in light of certain shortcomings. We collected retrospective data of clinical and biochemical parameters from in-hospital records. It was conducted at a single center and hence carries all the weaknesses of a single-center design. We were not able to capture the management strategies or the patient’s ability to take oral medication, which could also confound the length of stay. Moreover, the study was conducted in an adult population, thus inferences cannot be extrapolated in the pediatric age group, which holds a substantial percentage of the disease group. Since our hospital structure is private and there is a service fee, patients may have influenced the discharge decision. Finally, as this was a cross-sectional study, a cause-effect relationship cannot be determined, but further prospective studies in lieu of newer antibiotic agents and home health services should be performed to confirm and better define the nature of this association between diabetes mellitus and LOS among patients with PN.

## Conclusions

This study shows that in patients with PN, DM, and hypertension, fever or TLC count are not independently associated with prolonged length of stay, however, an increasing age, high creatine levels, urinary symptoms, upper tract, and lower tract separately were independently associated with delayed hospital discharge beyond five days.
